# NEVIGRAV Study: A Case–Control Analysis on Changes in Melanocytic Nevi During Pregnancy

**DOI:** 10.1111/1346-8138.17848

**Published:** 2025-07-07

**Authors:** Vincenzo Maione, Anna Venturuzzo, Stefano Bighetti, Carola Romanó, Zeno Fratton, Enzo Errichetti, Marina Venturini, Luca Bettolini

**Affiliations:** ^1^ Dermatology Department University of Brescia, ASST Spedali Civili di Brescia Brescia Italy; ^2^ Dermatology Department University of Udine Udine Italy

**Keywords:** Dermoscopy, nevi, pregnancy

## Abstract

Pregnancy can influence modifications and appearance of melanocytic nevi, but studies report inconsistent findings, often lacking standardized methods and comparison groups. This study evaluates dermoscopic changes in nevi and the development of new melanocytic lesions in pregnant women using full‐body photography and digital dermoscopy. Nevigrav is an observational case–control study conducted at two university dermatology centers, involving 85 participants (45 pregnant women and 40 controls) with 1614 lesions analyzed. Participants were asked about recent sun exposure or tanning habits. Enrollment was predominantly conducted in autumn and winter months to minimize ultraviolet (UV) exposure as a confounding factor. Total body photography revealed no new lesions in pregnant women when compared to controls. Dimensional growth was observed in the abdomen (*p* = 0.049) and the back (*p* < 0.001) between the first and second trimesters, with further abdominal growth in the third trimester (*p* < 0.001). At the third trimester, pigmentation changes were significant, with phototype III maintaining or increasing pigmentation and phototype II showing a reduction of pigmentation (*χ* ^2^ = 32.33, *p* < 0.001). Regional pigmentation differences were noted in the mammary (*p* = 0.02), abdominal (*p* = 0.004), and thigh (*p* = 0.007) areas. No changes in symmetry or dermoscopic nevi patterns, including dots or globules, were observed. Pregnancy does not increase nevi count but may cause size and pigmentation changes, particularly in the abdomen, influenced by phototype and body region. No new dermoscopic patterns or symmetry changes were found.

## 1 Introduction

During pregnancy, the levels of beta and alpha melanocyte‐stimulating hormones increase, along with elevated levels of estrogen, progesterone, and beta‐endorphin. These hormonal changes are believed to heighten skin pigmentation. Additionally, pregnant women frequently experience changes in the appearance of their melanocytic nevi, as well as the development of new nevi during pregnancy [1, 2]. The studies available in the literature on this topic often show differing results and employ different methodologies, frequently lacking a comparison group.

The aim of this multi‐center, prospective, longitudinal cohort study is to describe the dermoscopic changes in preexisting nevi and to assess the emergence of new melanocytic lesions in pregnant women using full‐body photography and digital dermoscopic follow‐up.

## 2 Methods

The study enrolled participants from the outpatient clinic of the Dermatology Department of two University Hospitals in northern Italy between September 2023 and May 2024. Participants were asked about sun exposure or tanning beds. Enrollment was predominantly conducted in the autumn and winter months to minimize ultraviolet (UV) exposure as a confounding factor.

The case group included women in their first trimester of pregnancy, whereas the control group included women of childbearing age without previous pregnancy. Patients with a history of melanoma were excluded in both groups to avoid bias. The visits took place during the first, second, and third trimesters of pregnancy (baseline, T1 and T2, respectively). Similarly, the control group underwent clinical and dermoscopic evaluations at comparable time intervals, matching the schedule of the pregnant patients as closely as possible.

Full‐body images were captured using a Canon‐Canfield Instellistudio. All melanocytic lesions ≥ 1 mm were registered with a portable wireless digital videodermatoscope (Demetra Scope). Dimensional assessments were conducted automatically using the software integrated into the videodermatoscopy system (Barco Demetra Analytics Toolkit). The automated Demetra analytics provided measurements for changes in nevi's size and shape, calculating major and minor diameters, area, and symmetry axes. We considered a change in nevus size to be significant only if it exceeded 5% compared to the baseline measurement. Variations equal to or less than 5% were considered within the margin of measurement error and categorized as “no change.”

Pigmentation changes were assessed through side‐by‐side comparison of baseline and follow‐up dermoscopic images. Changes were classified as either hyperpigmentation (increased pigmentation) or hypopigmentation (decreased pigmentation).

To ensure consistency and minimize variability in color representation, all dermoscopic images were acquired by the same investigator using the same device and under standardized conditions for lighting, magnification, and interface modality.

Moreover, all images were subsequently reviewed on a single monitor under the same viewing settings. This approach was adopted to avoid potential discrepancies in color perception due to differences in display calibration or ambient lighting.

Symmetry was defined as the regular and bilateral distribution of dermoscopic structures along one or two perpendicular axes, in accordance with the criteria of the Three‐Point Checklist.

All lesions were independently evaluated by two experienced dermoscopists. In cases of disagreement regarding either pigmentation or symmetry, a third board‐certified dermatologist with recognized expertise in dermoscopy performed an independent review to provide the final classification. If any lesions were identified as suspicious for neoplasia, standard clinical practice would be followed, including the excision of the lesion and subsequent histological evaluation. During the visit, demographic and clinical data were collected, including age, Fitzpatrick skin type, and personal and family history of skin cancers.

The pseudonymized data collection database was formatted using Microsoft‐Excel software and subsequently imported into IBM‐SPSS ver. 29.0.2. The use of Stata ver. 17.0 was also considered for comparisons or implementing test outputs.

Non‐parametric repeated‐measures analysis was performed using the Friedman test, followed by pairwise comparisons between time points using the Wilcoxon signed‐rank test.

The sample size was calculated based on a two‐sided t‐test for independent means. It was hypothesized that pregnant women would have a mean nevus area of 10 mm^2^ (±7 SD), compared to 6 mm^2^ (±5.5 SD) in non‐pregnant controls. This corresponds to an estimated effect size (Cohen's *d*) of approximately 0.65. To achieve 80% power with a significance level of *α* = 0.05, a minimum of 40 participants per group (80 total) was required.

The local ethics committee approved the study (NEVIGRAV NP 6248), and all participants provided written informed consent.

## 3 Results

The study involved 85 participants, of which 45 were pregnant women in the case group, and 40 were women of childbearing age in the control group. Demographic, clinical, and dermoscopy baseline characteristics are reported in Table 1. In the case group, the median age at baseline was 28.1 years (range: 25–41), while in the control group, it was 30 years (range: 27–41). The median gestational week at baseline, T1, and T2 was 12.50, 20.21, and 29.29 weeks, respectively, with minimum values of 6.29, 13.71, and 23.71 weeks, and maximum values of 16.14, 26.71, and 36.14 weeks, respectively. Regarding skin type, among the control group, skin type III was the most represented (60%). On the other hand, in the case group, skin type II was more prevalent (66.7%). No patients in either group had skin type IV, and only one control patient had skin type I. The familial history of melanoma showed consistent patterns across both populations. Notably, 80.0% of controls and 88.9% of cases did not exhibit this risk factor.

**TABLE 1:** Demographic, clinical, and dermoscopy baseline characteristics.

Baseline characteristics	Cases (*n* = 45)	Controls (*n* = 40)
Age (year), median (min‐max)	28.1 (25–41)	30 (27–41)
Fitzpatrick skyn type, *n* (%)		
I	0	1 (2.5)
II	30 (66.7)	15 (37.5)
III	15 (33.3)	24 (60)
Familiarity for melanoma, *n* (%)		
No	40 (88.9)	32 (80)
Yes	5 (11.1)	8 (20)
Gestational weeks, median (min‐max)		
Baseline	12.5 (6.3–16.1)	
T1	20.2 (13.7–26.7)	
T2	29.3 (23.7–36.1)	

	Cases (*n* = 801)	Controls (*n* = 813)
Number of nevi per patient, median (min‐max)	14 (3–43)	17 (3–45)
Larger diameter (mm), median (min‐max)	3.9 (1.5–17.4)	3.6 (1–18.2)
Area (mm^2^), median (min‐max)	8.4 (1.5–105.6)	6.9 (1.1–127.5)
Sites, *n* (%)		
Upper limbs	140 (17.5)	180 (22.1)
Lower limbs	157 (19.6)	186 (22.9)
Head and neck	13 (1.6)	32 (3.9)
Trunk	228 (28.5)	168 (20.7)
Back	263 (32.8)	247 (30.4)
Nevus type, *n* (%)		
Acquired	717 (89.5)	675 (83)
Medium Congenital	3 (0.4)	6 (0.7)
Congenital	48 (6)	60 (7.4)
Atypical	33 (4.1)	14 (1.7)
Shape symmetry, *n* (%)		
Yes	796 (99.4)	811 (99.7)
No	5 (0.6)	2 (0.3)
Dermoscopy pattern, *n* (%)		
Globular	21 (2.6)	80 (9.8)
Reticular	727 (90.8)	613 (75.4)
Homogenous	22 (2.7)	60 (7.4)
Cobblestone	16 (2)	27 (3.3)
Multicomponent	6 (0.7)	27 (3.3)
Parallel	5 (0.6)	1 (0.1)
Fibrillar	3 (0.4)	2 (0.2)
Lattice‐like	1 (0.1)	3 (0.4)
Dermoscopy features		
Peripheral dots, *n* (%)		
Absent	787 (98.3)	794 (97.7)
Regular	9 (1.1)	13 (1.6)
Irregular	5 (0.6)	6 (0.7)
Peripheral globules, *n* (%)		
Absent	775 (96.8)	763 (93.8)
Regular	23 (2.9)	47 (5.8)
Irregular	3 (0.4)	3 (0.4)
Streaks, *n* (%)		
Absent	801 (99.9)	813 (100)
Present	1 (0.1)^a^	0 (0)
Blotches, *n* (%)		
Absent	795 (99.3)	811 (99.8)
Present	6 (0.7)	2 (0.2)
Blue‐white veil, *n* (%)		
Absent	801 (99.9)	813 (100)
Present	1 (0.1)^a^	0 (0)

^a^ The nevus was excised after the baseline visit and excluded from analysis.

The two populations were analyzed for melanocytic nevi, with 813 in the control group and 801 in the cases, for a total of 1614 lesions (Table 1). In the control group, a median of 17 melanocytic nevi were documented per patient (range: 3–45). Meanwhile, in the cases, there were 14 melanocytic nevi per woman (range: 3–43). In terms of nevus measurements, the median larger diameter was 3.6 mm (range: 1–18.2) and 3.9 mm (range: 1.5–17.4) for controls and cases, respectively.

In terms of area, the baseline median was 6.9 mm^2^ (range: 1.1–127.5) for the control group and 8.4 mm^2^ (range: 1.5–105.6) for the case group. The distribution of melanocytic nevi across body regions differed between the control and case groups. The back was the most common site in both groups, accounting for 32.8% of lesions in the control group and 30.4% in the case group. The anterior part of the trunk followed, representing 28.5% of lesions in the control group and 20.7% in the case group. For the extremities, the upper limbs comprised 17.5% of nevi in the control group and 22.1% in the case group, while the lower limbs accounted for 19.6% and 22.9%, respectively. The abdominal area showed a notable difference, with 11.7% of control group lesions compared to 18% in the case group. The head and neck region had the lowest representation, comprising only 1.6% of lesions in the control group and 3.9% in the case group.

The majority of recorded nevi were acquired, accounting for 89.5% in the case group and 83% in the control group. Small congenital nevi were the second most common type, representing 6% in the case group and 7.4% in the control group. Clinical atypical nevi were more prevalent in the case group compared to the control group (4.1% vs. 1.7%). Medium congenital nevi were rare, making up only 0.4% in the case group and 0.7% in the control group.

In the control group, 811 nevi (99.8%) exhibited a symmetrical shape, compared to 796 nevi (99.4%) in the case group. Only a small percentage of nevi were asymmetrical, with 2 nevi (0.2%) in the control group and 5 nevi (0.6%) in the case group.

Table 1 also delineates the various baseline dermoscopic patterns observed in nevi—reticular, globular, homogeneous, cobblestone, multicomponent, parallel, lattice‐like, and fibrillar. The reticular pattern emerged as the predominant pattern, documented in 75.4% of the control group and 90.8% of the cases. This was succeeded by the globular pattern, seen in 9.8% of controls and 2.6% of cases, the homogeneous pattern in 7.4% of controls and 2.4% of cases, and the multicomponent pattern in 3.3% of controls versus 0.4% of cases. The residual patterns, as shown in Table 1, are less prevalent in both the case and control groups. When examining baseline dermoscopic features in nevi, regular and irregular peripheral dots were present in 1.6% and 0.7% of control lesions, and 1.1% and 0.6% of case lesions, respectively. Regular and irregular peripheral globules were noted in 5.8% and 0.4% of control lesions, and 2.9% and 0.4% of case lesions, respectively. Streaks or blue‐white veil patterns were absent in controls, while one case exhibited streaks and blue‐white veil patterns. The nevus in question was excised, and histopathological analysis confirmed an atypical nevus. Blotches were detected in 0.24% of control lesions and 0.75% of case lesions. Pertaining to the emergence of new nevi, only a single patient in the control group developed a new nevus on the left upper arm.

A Pearson's chi‐squared test shown in Table 2 revealed that modifications in shape, pattern, and/or features during at least one follow‐up visit were significantly more prevalent in cases compared to controls (7.3% vs. 2.7%, respectively; *p* < 0.001). Between T1 and T2, these modifications were notably higher in cases at every follow‐up visit (*p* < 0.001 for both). Conversely, familial melanoma history bore no significance.

**TABLE 2:** Statistical analysis on dimensional changes.

Variables	Cases (*n* = 2403)	Controls (*n* = 2439)	*X* ^2^	*p*
≥ 1 modification^a^, *n* (%)			52.44	< 0.001^a^
Yes	175 (7.3)	67 (2.7)		
No	2228 (92.7)	2372 (97.3)		
≥ 1 modification^a^, *n* (%)	Cases (*n* = 801)	Controls (*n* = 813)	*χ* ^2^	*p*
Baseline‐T1			15.05	< 0.001^a^
Yes	80 (10)	40 (4.9)		
No	721 (90)	773 (95.1)		
T1‐T2			42.10	< 0.001^a^
Yes	95 (11.9)	27 (3.3)		
No	706 (88.1)	786 (96.7)		
Area changes (mm^2^), *n* (%)			314.27	< 0.001^a^
Baseline‐T1			172.4	< 0.001^a^
Increase	406 (50.7)	159 (19.6)		
Decrease	273 (34.1)	467 (57.4)		
No	122 (15.2)	187 (23)		
T1‐T2			38.02	< 0.001^a^
Increase	216 (27)	184 (22.6)		
Decrease	387 (48.3)	312 (38.4)		
No	198 (24.7)	317 (39)		

Body site (*n* = 801)	df	*χ* ^2^	Friedman test *p*	Post Hoc Wilcoxon test *p*	
				Baseline‐T1	T1‐T2
Head–neck	2	2.93	0.23	NA	NA
Breast	2	6.39	0.02^a^	0.7	0.02^a^
Abdomen	2	60.19	< 0.001^a^	0.049^a^	< 0.001^a^
Back	2	26.69	< 0.001^a^	< 0.001^a^	< 0.001^a^
Upper limbs	2	38.21	< 0.001^a^	0.64	< 0.001^a^
Lower limbs	2	15.26	< 0.001^a^	0.45	< 0.001^a^
Area (mm^2^), median (min‐max)					

Body site, (*n* = 801)	Baseline	T1	T2
Head–neck	7.8 (3.5–45)	8.2 (3.6–45)	8.1 (3.5–45)
Breast	8.9 (1.5–60.5)	9.3 (2.1–62.6)	9 (1.5–65.7)
Abdomen	11.1 (1.5–65.3)	11.9 (1.5–64.9)	12.3 (1.5–62.9)
Back	9.4 (1.6–105.6)	9.9 (1.9–132.5)	9.7 (1.7–130)
Upper limbs	7.8 (2–25.4)	7.4 (2–24.3)	7.1 (2–25.9)
Lower limbs	6.6 (1.5–55.2)	6.7 (1.4–61.5)	6.7 (1.5–53.1)

Shape symmetry, *n* (%)	Cases (*n* = 801)		Controls (*n* = 813)		*χ* ^2^, *p*
	Yes	No	Yes	No	
Baseline	796 (99.4)	5 (0.6)	811 (99.7)	2 (0.3)	
T1	794 (99.1)	7 (0.9)	812 (99.9)	1 (0.1)	0.006, 0.63
T2	794 (99.1)	7 (0.9)	812 (99.9)	1 (0.1)	1.37, 0.12

^a^ Modification in shape, pattern, and/or features.

### 3.1 Dimensional Changes in Nevi During Pregnancy

Addressing changes in nevus dimensions outlined in Table 2, expressed as the area in mm^2^, 504 nevi in the control group (187 at T1 and 317 at T2) remained the same as the previous visit, while 343 increased (159 and 184, respectively) and 779 decreased (467 and 312, respectively). In contrast, the pregnant group had 320 nevi that remained unchanged (122 at T1 and 198 at T2), 622 that increased (406 at T1 and 216 at T2), and 660 that decreased (273 at T1 and 387 at T2). A chi‐square test demonstrated a differential in nevi dimension changes in the case group as opposed to the control group (*p* < 0.001). Furthermore, significant changes were consistent at T1 and T2 (*p* < 0.001 each). From baseline to T1 and T2, the median area of nevi in pregnant women was 8.4 (min‐max: 1.5–105.6), 8.6 (min‐max: 1.6–132.5) and 8.4 (min‐max 1.5–130), respectively. From the first to the second trimester of pregnancy, nevi were more likely to increase in size, while a decrease in dimensions was more pronounced during the third trimester.

**FIGURE 1:**
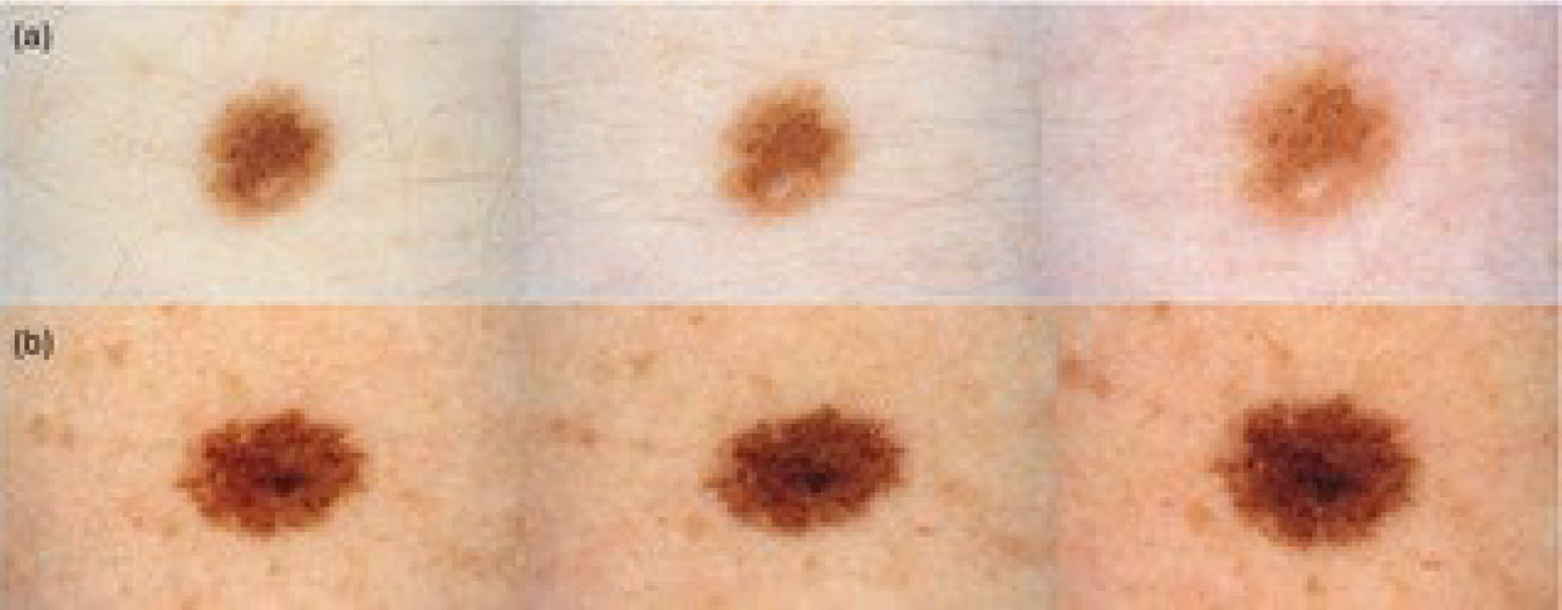
(a) Typical size increase of a nevus located at the abdominal level with final hypopigmentation (b) Hyperpigmentation of a nevus of the right thigh.

The global Friedman test, applied to all time points, revealed a significant difference among the area measurements (*χ* ^2^ = 54.73, *p* < 0.001). The Friedman test further analyzed changes in nevi size across time points, stratified by body region, for the pregnant group. Except for the head–neck region (*p* = 0.23), the results shown in Table 2 demonstrated a statistically significant difference in nevi dimensions across all other body regions, with *p* = 0.02 for the breast area and *p* < 0.001 for all remaining regions. Post hoc analysis revealed specific patterns of change across the time points. Significant differences were primarily observed between baseline and T1 in the abdomen (*p* < 0.001) and posterior trunk (including the back) (*p* = 0.049). Between T1 and T2, notable changes in nevi dimensions were detected, with *p* = 0.02 for the breast area and *p* < 0.001 for all other regions analyzed. Table 2 presents the median area with interquartile ranges stratified by body region. Based on interquartile data, an increase is observed in most of the sites considered. From the second to the third quarter, the only values to undergo a significant increase are those of abdominal nevi (Figure 1a).

In this area, the median increased from 11.1 at first trimester to 12.3 at third trimester, reflecting an enlargement of nevi concurrent with overall body growth over time. The shape symmetry of the lesions did not exhibit statistically significant changes at T1 and T2, in neither the cases nor the controls (*p* = 0.63 and *p* = 0.12, respectively).

### 3.2 Pigmentation Changes in Nevi During Pregnancy

In terms of pigmentation changes, at T1, 36 nevi (4.5%) in the case group and 26 nevi (3.2%) in the control group became hyperpigmented, while 48 (6%) and 42 (5.2%) became hypopigmented, respectively (Table 3). At T2, 38 in the cases (4.7%) and 9 in the controls (1.1%) nevi became hyperpigmented, and 67 (8.4%) and 19 (2.3%) became hypopigmented, respectively. A significant difference was observed in pigmentation changes at T2 (*p* < 0.001). An investigation into a possible correlation between pigmentation changes and phototype revealed that phototype III tended to either maintain consistent pigmentation or become hyperpigmented (Figure 1b), while phototype II significantly hypopigmented (*χ* ^2^ = 32.33, *p* < 0.001).

**TABLE 3:** Statistical analysis on pigmentation and dermoscopy changes.

Pigmentation changes, *n* (%)	Cases (*n* = 801)	Controls (*n* = 813)	*χ* ^2^	*p*
Baseline‐T1			2.52	0.282
Increase	36 (4.5)	26 (3.2)		
Decrease	48 (6)	42 (5.2)		
T1‐T2			60.91	< 0.001*
Increase	38 (4.7)	9 (1.1)		
Decrease	67 (8.4)	19 (2.3)		
T1‐T2			Increase	Decrease
Body site	*X* ^2^	*p*	*p*	*p*
Head–neck	0.9	0.64	NA	NA
Breast	8.1	0.02*	0.23	0.08
Abdomen	11.1	0.004*	0.617	0.004*
Back	2.52	0.24	NA	NA
Upper limbs	4.11	0.13	NA	NA
Lower limbs	9.8	0.007*	0.004*	1

Peripheral dots, *n* (%)	Cases (*n* = 801)			*χ* ^2^	*p*
	Baseline	T1	T2		
Absent	787 (98.3)	783 (97.9)	786 (98.1)	0.41	0.98
Present	14 (1.7)	17 (2.1)	15 (1.9)		

Peripheral dots, *n* (%)	Cases (*n* = 801)	Controls (*n* = 813)	*χ* ^2^	*p*	Cases (*n* = 801)	Controls (*n* = 813)	*χ* ^2^	*p*
	Baseline‐T1							
Increase	5 (3.5)	2 (0.2)	2.38	0.30	1 (0.1)	1 (0.1)	2	0.4
Decrease	3 (2)	1 (0.1)			4 (2.5)	1 (0.1)		

Peripheral globules, *n* (%)	Cases (*n* = 801)			*χ* ^2^	*p*
	Baseline	T1	T2		
Absent	775 (96.8)	776 (97)	773 (96.5)	0.49	0.97
Present	26 (3.2)	25 (3)	28 (3.5)		

Peripheral globules, *n* (%)	Cases (*n* = 801)	Controls (*n* = 813)	*χ* ^2^	*p*	Cases (*n* = 801)	Controls (*n* = 813)	*χ* ^2^	*p*
	Baseline‐T1							
Increase	4 (0.5)	8 (1)	2	0.09	1 (0.1)	2 (0.2)	2.6	0.27
Decrease	5 (0.6)	13 (1.6)			7 (0.9)	14 (1.7)		

*Note:* Significance of *indicates statistical significanceof *p* value when *p* < 0.05.

The analysis of modifications by body region at T2 showed significant differences between cases and controls in several locations. Specifically, we observed significant differences in the mammary (*p* = 0.02), abdomen (*p* = 0.004), and thigh (*p* = 0.007) regions when examining overall changes in pigmentation.

Further breakdown into pigmentation and within each significant region yielded nuanced insights. For the thigh region, only the increase in pigmentation was statistically significant (*p* = 0.004), while for the abdomen region, only the decrease reached significance (*p* = 0.004). In the mammary region, although a significant overall difference was initially found, neither increase (*p* = 0.08) nor decrease (*p* = 0.23) was individually significant, suggesting that the observed effect may be due to a general shift in pigmentation distribution rather than a distinct change in hyperpigmentation or depigmentation alone. No significant differences in pigmentation changes were observed in the other body regions.

### 3.3 Dermoscopic Changes in Nevi During Pregnancy

The reticular pattern, the most frequently reported in this study, formed by the pigmented network did not exhibit signs of asymmetrical growth, as shown by previous data, and followed the pigmentation trends reported earlier. Considering the other dermoscopic patterns outlined in Table 3, Pearson's chi‐squared test indicated no statistical difference in the emergence of nevi with dots, the disappearance of dots, or the modification of dots expressed as an increase or decrease at every follow‐up visit (*p* = 0.98, pT1 = 0.30, and pT2 = 0.4, respectively). A similar lack of significance was demonstrated considering peripheral globules (*p* = 0.97, pT1 = 0.09, and pT2 = 0.27). The other dermoscopic features were too infrequent to be considered statistically significant.

## 4 Discussion

Over the years, various studies have attempted to evaluate modifications in nevi during pregnancy, and despite some controversies, the results of this work estimate changes between 10.5% and 32.5% of cases [3, 4]. However, these studies employed varying methodologies, some of which were susceptible to subjective interpretation and were conducted without a control group. For this reason, the objective of our study was to assess all melanocytic lesions throughout pregnancy in a case–control study. As a primary objective, we evaluated the onset of new melanocytic lesions during the first, second, and third trimesters with standardized photography. In the literature, only the study by Martins‐Costa et al. has evaluated the appearance of new nevi during pregnancy [5]. In this work, 44% of pregnant patients showed the development of new lesions. Several body sites were involved, but all patients with new lesions had at least one on the upper limbs. Our study did not observe an increase in lesions through total body photography. It should be noted that the study by Martins‐Costa involved a younger population compared to ours, which might partially explain this increase in lesions. However, it is also important to highlight that the method used for calculating new nevi may lead to an overestimation compared to other technique [6] and the absence of an active comparator may affect the reliability of this finding. Regarding changes in lesion size, the result of our study aligns with current literature, which, although based on a smaller number of nevi examined, confirms this trend.

For instance, Aktruk et al. in a study of 97 nevi, observed an increase in size predominantly in the anterior regions of the body [7]. On the other hand, several studies in the literature, having established that nevi in anterior regions tend to change, have focused exclusively on dorsal lesions. In this context, both Perroyer's and Zampino's studies reported minimal size changes that were not statistically significant [8, 9]. Across all studies, including our own, dimensional changes were predominantly symmetrical.

Our study partially supports the hypothesis that dimensional changes can be attributed to cell growth, as proposed by Lee [10]. In fact, our data show dimensional growth in almost all the areas considered during baseline and T1. However, such growth is confirmed only at the abdominal level in the third trimester, where growth induced by physiological streaking is evident.

Another point of interest is the color changes in pigmented lesions during pregnancy. In the literature, only a few studies examine pigmentation comprehensively throughout pregnancy and the postpartum period. Zampino et al. reported a progressive reduction in lesion pigmentation over the course of pregnancy [9]. Conversely, in another study, Rubegni et al. observed an increase in nevi contrast during pregnancy, with a return to normal appearance only 12 months postpartum [11]. Finally, Martins‐Costa's study documented hyperpigmentation in 10.4% of cases and hypopigmentation in 5.8%, though this data was not explored further.

In our study, pigment changes became noticeable only between the sixth and ninth months, showing a dichotomy: some patients tended toward hyperpigmentation, while others tended toward hypopigmentation. Analyzing this data by lesion location, it became evident that breast lesions exhibited both types of pigment changes; abdominal lesions tended to hypopigment, while lower limb lesions tended to hyperpigment. Moreover, a further stratification by phototype revealed that patients with darker skin tones tended to hyperpigment, while those with phototypes I and II showed a trend toward hypopigmentation. This finding is particularly noteworthy given the existing literature. Indeed, the hypothesis that estrogen activity indiscriminately increases pigmentation is not supported by our study [12]. Furthermore, on the abdomen—where skin pigmentation generally darkens (e.g., abdominal linea nigra)—nevi instead tended to hypopigment.

Moreover, it is important to mention that in our study, assessments were scheduled closer to the end of the first trimester, meaning that earlier changes could not have been recorded.

From a dermatoscopic perspective, various authors have focused primarily on the appearance of new structures or modifications to existing ones.

In this work, no increase or reduction in globules or dots was observed, nor was there any appearance or disappearance of these structures. It should be noted that the literature on this topic presents varying findings: Akturk, Zampino, and Martins‐Costa report either an increase in globules or hyperpigmentation of these features. The emergence or modification of this pattern is often a sign of nevus growth and is generally associated with younger individuals. In our study, the age of pregnant participants was higher compared to other studies, reflecting a common demographic trend in Italy, and this factor likely explains the discrepancy with existing literature. However, it should be emphasized that the absence of this pattern further supports our findings that enlargement is not due to mere replicative activity of the nevi.

In conclusion, our work confirms that, during pregnancy, an increase in the size of nevi may occur in different body regions between the first and second trimesters, while in the third trimester, the dimensional growth of these lesions appears to be sustained only in the abdomen, an area subject to normal tissue expansion during the gestational process.

In the majority of cases, these changes, though involving color intensity shifts, remain symmetrical. Dermoscopically, these lesions do not exhibit substantial alterations and for this reason, asymmetric changes should still be seen as suspicious. Therefore, with regard to the monitoring of nevi during pregnancy, it is possible to adopt an inverse reasoning approach: given the symmetrical growth of lesions on the chest and abdomen, changes occurring in other areas of the body, especially if they exhibit asymmetry, should be carefully monitored in pregnant patients. It should be noted that these data should be confirmed in a larger studies.

However, our study has some limitations that should be highlighted, mainly due to the absence of pre‐ and post‐pregnancy evaluations of the nevi. For this bias, identifying women who will become pregnant within a case–control study design represents a methodological challenge, as it is difficult to anticipate such events prospectively.

On the other hand, while some studies have reported a return to baseline appearance of melanocytic lesions after delivery, this could not be confirmed in our cohort due to the lack of post‐partum follow‐up data. Most patients did not respond to our follow‐up requests, likely due to the challenges and reduced availability commonly experienced in the immediate post‐pregnancy period.

An additional limitation of our study is the imbalance in phototype distribution between the group of pregnant patients and the control group. Specifically, phototype III individuals are more prevalent in the control group, and this should be taken into account when interpreting our findings, as skin phototype may influence dermoscopic features and skin responses. This imbalance could potentially confound the comparison between groups. However, it is important to note that the control group was selected entirely at random, and no matching for phototype was applied due to the exploratory nature of the study and the limited availability of eligible non‐pregnant controls during the enrollment period. Nonetheless, it is important to note that this work currently includes the largest number of monitored nevi and is the only one to provide tracking of pigmented lesions across all three trimesters, as well as a comparison group. Further investigations in this domain are warranted to validate the identified findings.

## Ethics Statement

Reviewed and approved by Brescia Ethics Committee.

## Consent

The patients in this manuscript have given written informed consent to publication of their case details.

## Conflicts of Interest

The authors declare no conflicts of interest.

## Data Availability

The data that support the findings of this study are available from the corresponding author, V.M., upon reasonable request.
